# Comprehensive analysis reveals CCDC60 as a potential biomarker correlated with prognosis and immune infiltration of head and neck squamous cell carcinoma

**DOI:** 10.3389/fonc.2023.1113781

**Published:** 2023-03-30

**Authors:** Zhixin Liu, Shuai Chen, Wenming Jia, Ye Qian, Xiaoqi Yang, Minfa Zhang, Tianhe Fang, Heng Liu

**Affiliations:** ^1^ Department of Orthopedics, Qilu Hospital of Shandong University, Jinan, Shandong, China; ^2^ NHC Key Laboratory of Otorhinolaryngology, Qilu Hospital of Shandong University, Jinan, Shandong, China; ^3^ Department of Otorhinolaryngology, Qilu Hospital of Shandong University, Jinan, Shandong, China; ^4^ Shandong University of Traditional Chinese Medicine, Jinan, Shandong, China

**Keywords:** CCDC60, hNSC, prognosis, immunotherapy, immune infiltration

## Abstract

**Background:**

Coiled-coil domain containing 60 (CCDC60) is a member of the CCDC family, which participates in the progression of many types of cancer. However, the prognostic value of CCDC60 in head and neck squamous cell carcinoma (HNSC) and its function in tumor immunity remain unclear.

**Methods:**

CCDC60 expression and its prognostic potential in HNSC were evaluated by bioinformatics approaches, which was validated in human HNSC samples. Genetic alteration analysis of CCDC60 and the underlying biological function of CCDC60 related co-expressed genes in HNSC were analyzed. The impact of CCDC60 on the regulation of immune infiltration in HNSC was comprehensively investigated. *In vitro*, a series of functional assays on CCDC60 were performed in HNSC cells.

**Results:**

Our study has indicated that compared with the adjacent normal tissues, CCDC60 expression was considerably downregulated in HNSC tissues. High CCDC60 expression was connected with favorable outcome of HNSC patients, and its prognostic significance was examined by distinct clinical characteristics. We identified the CCDC60-related co-expression genes, which were mainly enriched in the NOD-like receptor signaling pathway associated with the inhibition of tumor growth, leading to a better prognosis of HNSC patients. *In vitro*, CCDC60 overexpression significantly inhibited the growth, migration and invasiveness but regulated cell cycle progression, and promoted cell adhesion of Fadu and Cal27 cells. Additionally, high CCDC60 expression had strong connections with the infiltrating levels of immune cells, immune marker sets, immunomodulators and chemokines in HNSC, suggesting that targeting CCDC60 could be a promising strategy to enhance the efficacy of immunotherapy for HNSC patients.

**Conclusion:**

Tumor suppressor CCDC60 may be identified as a prognostic and immune-related indicator in HNSC, which had the potential functions in regulating the immune infiltration of HNSC and improving the response to immunotherapy for HNSC patients.

## 1 Introduction

Head and neck squamous cell carcinoma (HNSC) is notorious for its poor prognosis, with a 5-year survival rate of less than 50% (1). The most common risk factors reported are tobacco and alcohol, which account for 75% of HNSC (2). Although the combinations of surgery, chemotherapy, radiotherapy and immunotherapy have been applied, the outcome of advanced HNSC patients remains unsatisfactory (3, 4). Targeting of immune checkpoints, such as PD-1 and PD-L1, effectively improves the prognosis of cancer patients; However, only a small percentage of patients could benefit from it because of the irregularity in clinical response (5). Nowadays, considerable attentions are focused on searching for new therapeutic targets that predict response to immunotherapy of cancer. And effective biomarkers associated with prognosis and immunity, and better understandings of the mechanism related to immune regulation in HNSC are urgently required.

Coiled-coil domain containing (CCDC) protein family members took part in the occurrence and development of cancers, such as the invasion and metastasis of malignancies (6–8). As a new member, CCDC43 could mediate the differentiation and metastasis of gastric cancer (GC), and it was closely linked with the prognosis of GC patients (9). In non-small cell lung cancers, CCDC19 played an inhibitory role and effectively inhibited the tumor growth by targeting miR-184 (10). In addition, it has been reported that CCDC proteins had a great potential in modulating tumor immunity (11). As a predictor of poor prognosis, CCDC137 was found to be correlated with tumor immunosuppressive status and in lower grade glioma and uveal melanoma, CCDC137 could regulate the high infiltration levels of tumor associated macrophages (TAMs) and cancer associated fibroblasts (CAFs) (12). However, rarely reports have detected the function and underlying immune-related mechanism of CCDC60 in head and neck squamous cell carcinoma.

In our research, CCDC60 expression and its relation to HNSC patient prognosis were detected using bioinformatics methods, and validated by quantitative real-time polymerase chain reaction (qRT-PCR). Genetic mutation analysis of CCDC60 and its connection with prognosis in HNSC were examined by cBioPortal database. CCDC60 co-expression genes and its potential biological function as well as molecular mechanism were identified. We analyzed the impacts of CCDC60 expression on the growth, apoptosis, cell cycle, migration, invasion and adhesion of Fadu and Cal27 cells. Additionally, the connections between the expression of CCDC60 and tumor-infiltrating immune cells, immune marker sets, immunomodulators and chemokines in HNSC were explored. Our study suggested the important involvement of CCDC60 in HNSC, and the underlying mechanism by which CCDC60 regulated the immune infiltration of HNSC, which may provide new insights into immunotherapy for HNSC patients.

## 2 Materials and methods

### 2.1 Patients and specimens

Human HNSC samples were gained from patients undergoing surgery at Qilu Hospital of Shandong University, China. The patients were randomly selected and no one received any chemotherapy before surgery. A total of 23 HNSC-HSCC tissues and paired adjacent tissues (15 pairs without lymph node metastasis and 8 pairs with lymph node metastasis) were gained. In lymph node metastasis group, 7/8 patients had alcohol habit and 6/8 patients with tobacco smoking history. In no lymph node metastasis group, 13/15 patients with alcoholism and 12/15 with smoking habit. Patients’ detailed clinical information were shown in **Supplementary Material 1**. All patients signed informed consent and our research was approved by the Institutional Ethics Committee at Qilu Hospital.

### 2.2 Gene expression analysis of CCDC60

The differential expression of CCDC60 mRNA among TCGA tumors was analyzed *via* the TIMER2.0 database (13). P-value cutoff < 0.05 and the statistical significance was examined by Wilcoxon test. CCDC60 expression in HNSC samples was detected by TCGA database (n = 546) *via limma* package in R (**Supplementary Material 2**). The *pROC* R package was employed to plot the ROC curve of CCDC60 gene (14), and the predictive utility of CCDC60 for HNSC diagnosis was evaluated by the area under the curve (AUC). Values of AUC > 0.7 and P < 0.05 suggested that the gene had high predictive ability. The expression of CCDC60 in HNSC from different clinical characteristics was detected by the UALCAN database, including individual cancer stage, lymph node metastasis status, tumor grade, TP53 status, patient’s gender and age (15).

### 2.3 Quantitative real-time PCR (qRT-PCR)

Apply TRIzol reagent (Sigma-Aldrich) to gain total RNAs from HNSC tissues, then reversely transcribed to cDNA with the reverse transcription kit (TaKaRa). qRT-PCR was conducted by SYBR qPCR Master Mix (Thermo Fisher). The 2^−ΔΔCt^ method was utilized to quantify the relative mRNA expression, and ß-actin was selected as the inner control. The sequences of CCDC60 were as follows: CCTCTTCCGCCAGCTCTGTG (sense) and CACCCGGGTCCTTTGGGTTC (antisense). The sequences of ß-actin were as follows: CACCATTGGCAATGAGCGGTTC (sense) and AGGTCTTTGCGGATGTCCACGT (antisense).

### 2.4 Genetic alteration and survival analysis of CCDC60 in HNSC

The genetic mutation of CCDC60 and alteration-related prognosis in HNSC samples was detected *via* cBioPortal (16). Kaplan-Meier (KM) plotter was applied to determine the relation between CCDC60 expression and clinical prognosis of HNSC patients with distinct clinicopathological characteristics, including overall survival (OS) and relapse-free survival (RFS) (17). The OS and disease-specific survival (DSS) maps of CCDC60 gene in HNSC samples were obtained from the DriverDBv3 database (18).

### 2.5 Functional enrichment analysis of CCDC60 Co-expression genes in HNSC

By the Spearman correlation test, we identified CCDC60 co-expression genes in HNSC samples from TCGA dataset (n = 520) *via* the LinkedOmics database (19). KEGG pathway and GO enrichment analysis of these genes in HNSC samples are available at Gene Set Enrichment Analysis (GSEA). We used the GeneMANIA database to construct the protein-protein interaction (PPI) networks of CCDC60 related co-expression genes in HNSC samples (20).

### 2.6 Cell culture

Human FaDu and Cal27 cell lines were preserved in our lab. The cells were cultured in DMEM/MEM medium (BasalMedia) containing 10% fetal bovine serum (FBS) in a humidified atmosphere containing 5% CO_2_ at 37°C.

### 2.7 Plasmid and transfection

pENTER-CCDC60 overexpression plasmid was purchased from Vigenebio, and was transfected into Fadu and Cal27 Cells using Lipofectamine 3000 (Thermo Fisher), according to the manufacturer’s recommendations. 48 hours After transfection, cells were harvested or passaged for subsequent experiments.

### 2.8 Colony formation assay

Transiently transfected cells were maintained in culture medium for 14 days at 37°C. The colonies with more than 20 cells were counted. Fixed with 4% paraformaldehyde and stained with 0.1% crystal violet. Visible colonies were manually imaged and counted.

### 2.9 Cell counting kit-8 (CCK-8) assay

Approximately 5×10^3^ transfection cells were planted into 96-well plates and cultured for 24, 48, and 72 hours, respectively. After adding10 μL CCK-8 (Dojindo), the cells were incubated for 2 hours at 37°C in the dark. Finally, the value of optical density (OD) at 450 nm was measured by microplate reader.

### 2.10 Flow cytometric analysis

Approximately 1.5×10^5^ transfection cells were planted into 6-well plates and incubated for 48 hours. The cells were fully digested, harvested and washed twice with ice-cold phosphate buffer saline (PBS). Next, the cells were resuspended in binding buffer, and stained with 5 µL of fluorescein isothiocyanate (FITC) annexin V and propidium iodide (PI) for 15 minutes at 37°C in the dark. The results of apoptosis were analyzed by CytEpert v2.0 (Beckman Coulter).

For cell cycle analysis, the cell cycle detection kit (Bestbio) was selected. After transfection, Fadu and Cal27 cells were digested by trypsin, washed twice with PBS, fixed with 75% ice-cold ethanol and stored at -20°C for 1 hours. The cells were resuspended and stained with RNaseA reagent (50 U/ml) and propidium iodide (PI, 50 µg/mL) at 4°C for 30 minutes. Finally, cell cycle was determined by a flow cytometer (Beckman Coulter).

### 2.11 Wound-healing assay

After transfection, approximately 3×10^5^ cells were seeded into six-well plates. After 24 hours, scratch with a 10-ml micropipette tip in the center of the wells. Washed twice by PBS, the cells were incubated in 1% FBS medium. Scratch images were taken every 4 hours to compute the scratch healing area.

### 2.12 Transwell assay

Transwell chambers were utilized to examine the invasion ability of Fadu and Cal27 cells. Approximately 1×10^5^ cells were planted into each well. The upper chambers were contained in 200 µl serum-free medium, while the lower chambers in the medium containing 20% FBS. After 24 hours incubation, fixed the cells and stained with 0.1% crystal violet (Solarbio). Finally, the cells were photographed under a microscope, and stained cells were counted.

### 2.13 Cell adhesion assay

Cell adhesion assay was conducted using the cell adhesion kit (BestBio). Added 100 μL coating buffer into 96 well plates and incubated at 4°C overnight. Wash each well with washing solution, then approximately 5×10^4^ transfection cells were planted and incubated for 1.5 hours at 37°C. Washed with medium and added 100 μL fresh medium. Finally, the cells were stained with 10 μL staining solution B and incubated for 2 hours at 37°C, and the OD value at 450 nm was measured using a microplate reader (BioTek, Winooski, VT, USA).

### 2.14 Immune infiltration analysis of CCDC60 in HNSC

Applying TIMER2.0 database, we examined the connection between CCDC60 expression and the tumor-infiltrating lymphocytes (TILs) in HNSC, including CD4/CD8+ T cells, B cells, Tregs, Neutrophils, Macrophages, Monocytes, NK cells, Dendritic cells, and Mast cells. Next, the relation of CCDC60 expression with immune marker sets of immune cells was detected. These biomarkers of immune cells have been studied before (21–23). The analysis was based on the Spearman’s method and gene expression levels were displayed with log2 RSEM. Additionally, TISIDB database was developed to investigate the relationships of CCDC60 expression with immunostimulators, immunoinhibitors, chemokines, and receptors in HNSC (24).

### 2.15 Statistical analysis

R software (version 4.1.2) and GraphPad Prism7 were applied for data visualization and statistical analysis. The presence and strength of relationships between variables were determined by Spearman and Pearson correlation tests. The KM method was used to evaluate survival analyses. A ROC curve was plotted to examine the predictive performance of the CCDC60 risk score for HNSC diagnosis. The difference was statistically significant at p < 0.05.

## 3 Results

### 3.1 The expression of CCDC60 in HNSC

**Figure 1:**
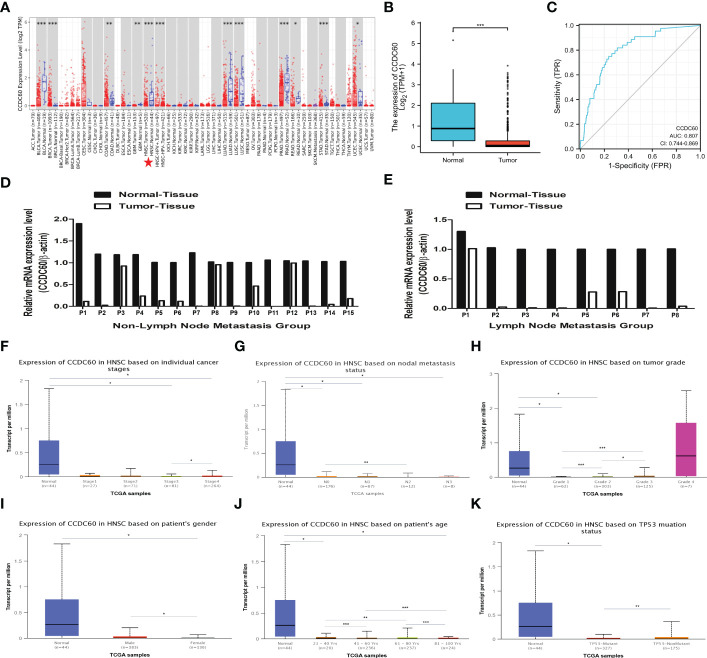
The expression level of CCDC60 in HNSC. **(A)** The mRNA expression of CCDC60 in different types of human cancers *via* the TIMER2.0 database. **(B)** Decreased CCDC60 expression in HNSC samples from the TCGA database (n= 546). **(C)** ROC curve analysis of CCDC60 gene for HNSC diagnosis. **(D)** CCDC60 mRNA expression levels in 15 pairs of HNSC tissues without lymph node metastasis **(E)** CCDC60 mRNA expression levels in 8 pairs of HNSC tissues with lymph node metastasis. Box-whisker plots showing the CCDC60 transcription in subgroups of HNSC patients, stratifed based on individual cancer stage **(F)**, node metastasis status **(G)**, tumor grade **(H)**, patient gender **(I)**, age **(J)** and TP53 mutation **(K)**. *P < 0.05, **P < 0.01, ***P < 0.001.

We realized that the expression level of CCDC60 mRNA was significantly downregulated in most types of cancers *via* the TIMER2.0 database (P < 0.05, **Figure 1A**). Compared with the adjacent normal tissues, CCDC60 expression was decreased in HNSC tissues from the TCGA database (n = 546), which was similar to the results of TIMER2.0 database (P < 0.001, **Figure 1B**). Analysis of ROC curve suggested that CCDC60 gene had a good predictive performance for HNSC diagnosis in the TCGA database (AUC = 0.807, **Figure 1C**). qRT-PCR results indicated that in 15 pairs of HNSC samples without lymph node metastasis, CCDC60 mRNA expression was lower than that in adjacent healthy tissues (**Figure 1D**). Similarly, in the 8 pairs of lymph node metastasis group, the consistent results were achieved (**Figure 1E**).

Based on the analysis of cancer stage, lymph node metastasis status, tumor grade, patients’ gender, age and TP53 status, CCDC60 expression was significantly downregulated in different subgroup of HNSC patients (P < 0.05, **Figures 1F–K**). Interestingly, in the subgroup of lymph node metastasis, CCDC60 expression was significantly lower in the N2 group than N0 group (P < 0.01, **Figure 1G**). In the tumor grade subgroup, we noticed the increasing expression of CCDC60 with increasing tumor grade in HNSC patients (**Figure 1H**). CCDC60 expression in male was significantly higher than female, and its expression was obviously different in various age subgroups (P < 0.05, **Figures 1l**, **J**). Additionally, CCDC60 expression was decreased in the TP53 mutant group compared with the nonmutant (P < 0.01, **Figure 1K**). We concluded that CCDC60 was significantly decreased in HNSC tissues and could be a promising indicator for HNSC identification and diagnosis.

### 3.2 CCDC60 was correlated with genetic alteration and better prognosis of HNSC patients

**Figure 2:**
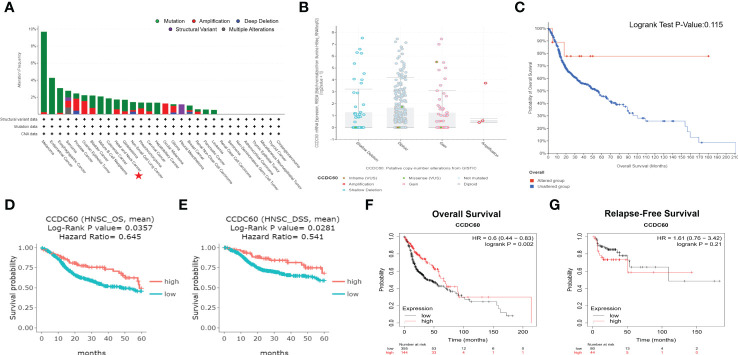
Genetic Alteration and Survival Analysis of CCDC60 in HNSC. **(A)** The alteration frequency with mutational types of CCDC60. **(B)** CCDC60 copy number variations in HNSC. **(C)** Survival analysis of CCDC60 altered group and unaltered group. **(D, E)** Survival curves of overall survival (OS) and disease-specific survival (DSS) of CCDC60 in HNSC patients from the DriverDBv3 database. **(F, G)** Survival curves of OS and relapse-free survival (RFS) of CCDC60 in the HNSC cohort by Kaplan-Meier plotter.

Analysis of genetic alteration showed that the alteration frequency of CCDC60 was 1.80% in 523 HNSC samples, including amplification (0.6%) and mutation (1.2%, **Figure 2A**). As shown in **Figure 2B**, the diploid was the most common copy number variations in HNSC patients. Between the altered and unaltered groups, difference in OS probability was not significant, suggesting that overall alteration of CCDC60 was not the cause of worse outcome led by low CCDC60 expression (P = 0.115, **Figure 2C**). Compared with the low group, high CCDC60 expression was significantly connected with longer OS (HR = 0.645, P = 0.0357) and DSS (HR = 0.541, P = 0.0281) of HNSC patients (**Figures 2D, E**). KM plotter demonstrated that CCDC60 expression was related to better OS (HR = 0.600, P = 0.002); however not with RFS (HR = 1.610, P = 0.210) (**Figures 2F, G**).

Based on the clinicopathological factors, we evaluated the relation between CCDC60 expression and clinical prognosis in HNSC. As shown in **Table 1**, we noticed that increased CCDC60 mRNA expression was linked with OS in male (HR = 0.560, P = 0.0024), white race (HR = 0.570, P = 0.0011) and tumor grade 3 (HR = 0.430, P = 0.0058). Specifically, upregulated CCDC60 expression was related to longer OS and RFS in stage 2 (OS, HR = 2.430, P = 0.0355; RFS, HR = 0.190, P = 0.0393), stage 3 (OS, HR = 0.460, P = 0.0416) and stage 4 (OS, HR = 0.480, P = 0.001) of HNSC patients. The above analysis indicated that CCDC60 expression was associated with a favorable outcome in HNSC, highlighting the function of CCDC60 in predicting the prognosis of HNSC, and the prognostic value of CCDC60 for HNSC patients was determined by its distinct clinical characteristics.

**Table 1:** Correlation of CCDC60 mRNA expression and clinical prognosis in head and neck squamous cell carcinoma with different clinicopathological factors by Kaplan–Meier plotter.

Clinicopathological characteristics	Overall survival			Relapse-free survival		
	N	Hazard ratio	P	N	Hazard ratio	P
Sex						
Female	164	0.76 (0.45 - 1.28)	0.3014	94	0.55 (0.18 - 1.64)	0.2764
Male	445	0.56 (0.38 - 0.82)	**0.0024**	94	1.58 (0.54 - 4.6)	0.3959
Race						
White	526	0.57 (0.41 - 0.8)	**0.0011**	133	0.58 (0.25 - 1.31)	0.1831
Asian	11	–	–	4	–	–
Black/African America	74	0.66 (0.27 - 1.66)	0.3794	5	–	–
Stage						
1	50	0.23 (0.02 - 2.26)	0.1711	66	4.06 (0.74 - 22.3)	0.082
2	91	2.43 (1.03 - 5.71)	**0.0355**	57	0.19 (0.03 - 1.12)	0.0393
3	98	0.46 (0.21 - 0.99)	**0.0416**	49	1.92 (0.5 - 7.44)	0.3356
4	308	0.48 (0.31 - 0.75)	**0.001**	0	–	–
Grade						
1	84	0.41 (0.09 - 1.85)	0.2334	36	4.56 (0.81 - 25.7)	0.0608
2	340	0.69 (0.45 - 1.05)	0.0798	78	0.37 (0.13 - 1.07)	0.0571
3	158	0.43 (0.23 - 0.8)	**0.0058**	49	0.23 (0.03 - 1.92)	0.1381
4	7	–	–	1	–	–
Mutation burden						
High	307	0.76 (0.51 - 1.15)	0.1978	99	0.5 (0.11 - 2.4)	0.3811
Low	296	0.53 (0.35 - 0.79)	**0.0019**	89	0.49 (0.19 - 1.25)	0.1262

Bold values indicate p < 0.05. –, None.

### 3.3 Gene set enrichment analysis (GSEA) of CCDC60 related co-expressed genes in HNSC

**Figure 3:**
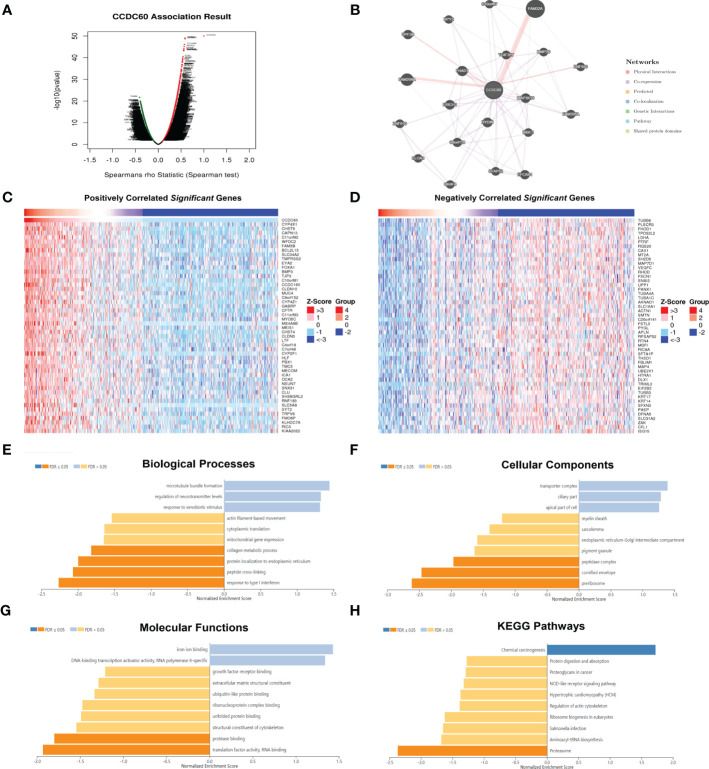
Functional analysis of CCDC60 related co-expressed genes in HNSC. **(A)** The volcano plot of CCDC60 co-expression genes. The red dots represented genes that are positively related to CCDC60, while the green dots are the opposite. **(B)** Protein-protein interaction (PPI) network of CCDC60-related genes by the GeneMANIA databse. **(C, D)** The top 50 significantly positive or negative genes which linked to CCDC60 in HNSC. Functional analysis of CCDC60 co-expression genes in HNSC samples are available at the LinkedOmics database, including Biological processes, BP **(E)**; Cellular components, CC **(F)**; Molecular functions, MF **(G)**; KEGG pathway **(H)**.

We have identified the CCDC60 related co-expression genes using the LinkedOmics database [FDR(BH) < 0.001, **Supplementary Material 3**], among which 5520 genes were positively linked with CCDC60 and 1688 genes were negatively correlated (**Figure 3A**). The PPI network elaborated the potential connections between CCDC60 related co-expression genes (**Figure 3B**). **Figures 3C**, **D** indicated the top 50 genes positively or negatively correlated with CCDC60 in HNSC. GO analysis suggested that these genes were mainly involved in the peptide cross-linking, peptidase complex, protease binding and translation factor activity, RNA binding (**Figures 3E–G**). KEGG pathway indicated enrichment in the NOD-like receptor signaling pathway, associated with chemical carcinogenesis (**Figure 3H**). The above analysis revealed the potential function of CCDC60 in HNSC, which CCDC60 might act as a tumor suppressor that inhibited the HNSC progression through sphingolipid signaling pathway.

### 3.4 CCDC60 inhibited the proliferation, regulated cell cycle progression of Fadu and Cal27 cells

**Figure 4:**
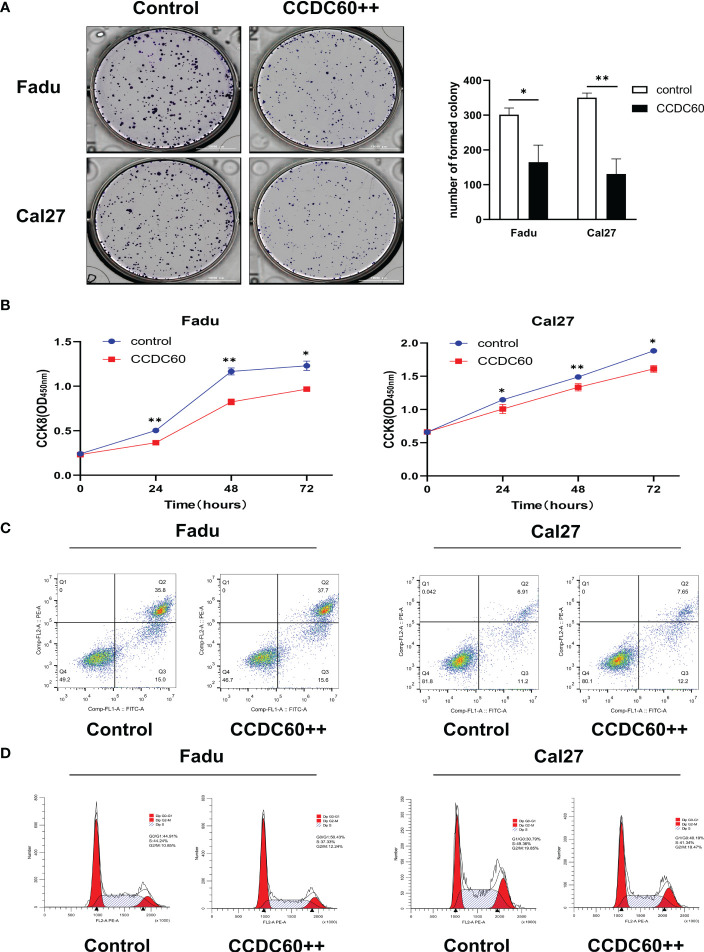
CCDC60 inhibited the proliferation, regulated cell cycle progression of HNSC cells. **(A)** Colony formation assay and the related analysis in Fadu and Cal27 cells treated as indicated. **(B)** Growth curves of Fadu and Cal27 cells with indicated treatment were examined by CCK-8 assay. **(C)** Apoptotic rate of Fadu and Cal27 cells with indicated treatment were detected *via* flow cytometry. **(D)** The cell cycle distribution of Fadu and Cal27 cells treated as indicated. *P < 0.05, **P < 0.01.

pENTER-CCDC60 overexpressed plasmid was successfully transfected (**Figure S1**). In colony formation assay, we realized that compared with the control group, both Fadu and Cal27 cells overexpressing CCDC60 formed fewer clones (P < 0.05, **Figure 4A**). Consistently, the proliferation abilities of both cell types were significantly attenuated after pENTER-CCDC60 plasmid transfection at 24, 48 and 72 hours in the CCK-8 assay (P < 0.05, **Figure 4B**). Since dysfunction of chromosome formation results in mitosis failure and apoptosis, to identify the mechanism by which CCDC60 affects the tumorigenesis of HNSC, we detected the influences of CCDC60 on apoptosis and cell cycle progression. By the flow cytometry, we found that there was no significant difference in the rate of apoptosis between these groups (P > 0.05, **Figure 4C**). CCDC60 overexpression increased the proportion of cell cycle arrest in the G0-G1 phases, while the number of cells in the S-phase was obviously decreased (**Figure 4D**). These results showed that CCDC60 overexpression could inhibit the proliferation, result in G0-G1 cell cycle transition arrest in HNSC.

### 3.5 CCDC60 suppressed the migration and invasion, promoted cell adhesion of Fadu and Cal27 cells

**Figure 5:**
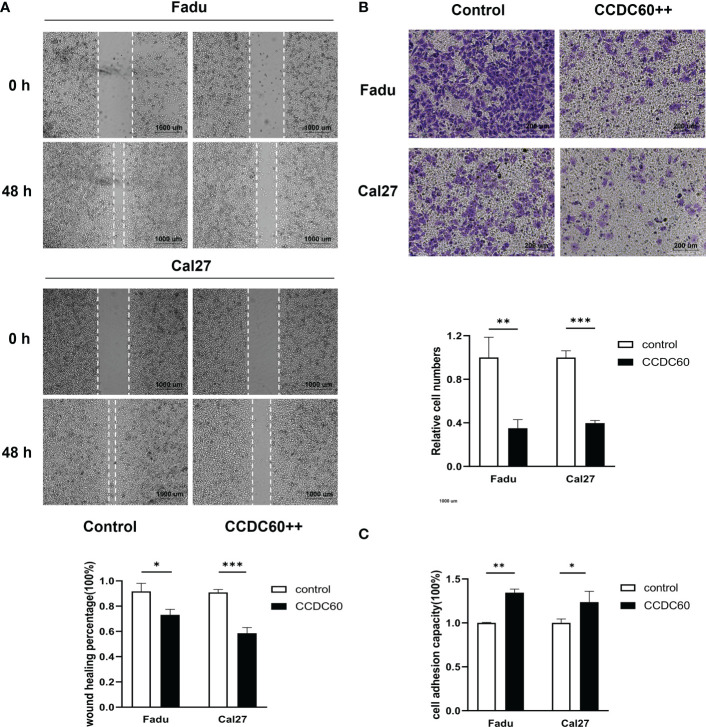
CCDC60 suppressed the migration and invasion, promoted cell adhesion of HNSC cells. **(A)** Wound-healing assay and the related analysis of Fadu and Cal27 cells treated as indicated. **(B)** Transwell assay and the related analysis of Fadu and Cal27 cells with indicated treatment. **(C)** Cell adhesion assay and the related analysis of Fadu and Cal27 cells treated as indicated. *P < 0.05, **P < 0.01, ***P < 0.001.

The findings of wound-healing and transwell assays showed that compared to that of control, overexpression of CCDC60 significantly inhibited the migration of Fadu and Cal27 cells (P < 0.05, **Figure 5A**), and the number of invading pENTER-CCDC60 transfected cells was markedly decreased (P < 0.01, **Figure 5B**). In cell adhesion assay, we observed the number of adhesion cells treated with pENTER-CCDC60 plasmid was higher than that in the control group (P < 0.05, **Figure 5C**). The meaningful results suggested that CCDC60 could markedly reduce the migration and invasive activities, while promote the capacity to cell adhesion in HNSC.

### 3.6 CCDC60 expression was closely related to immune infiltration of HNSC

**Figure 6:**
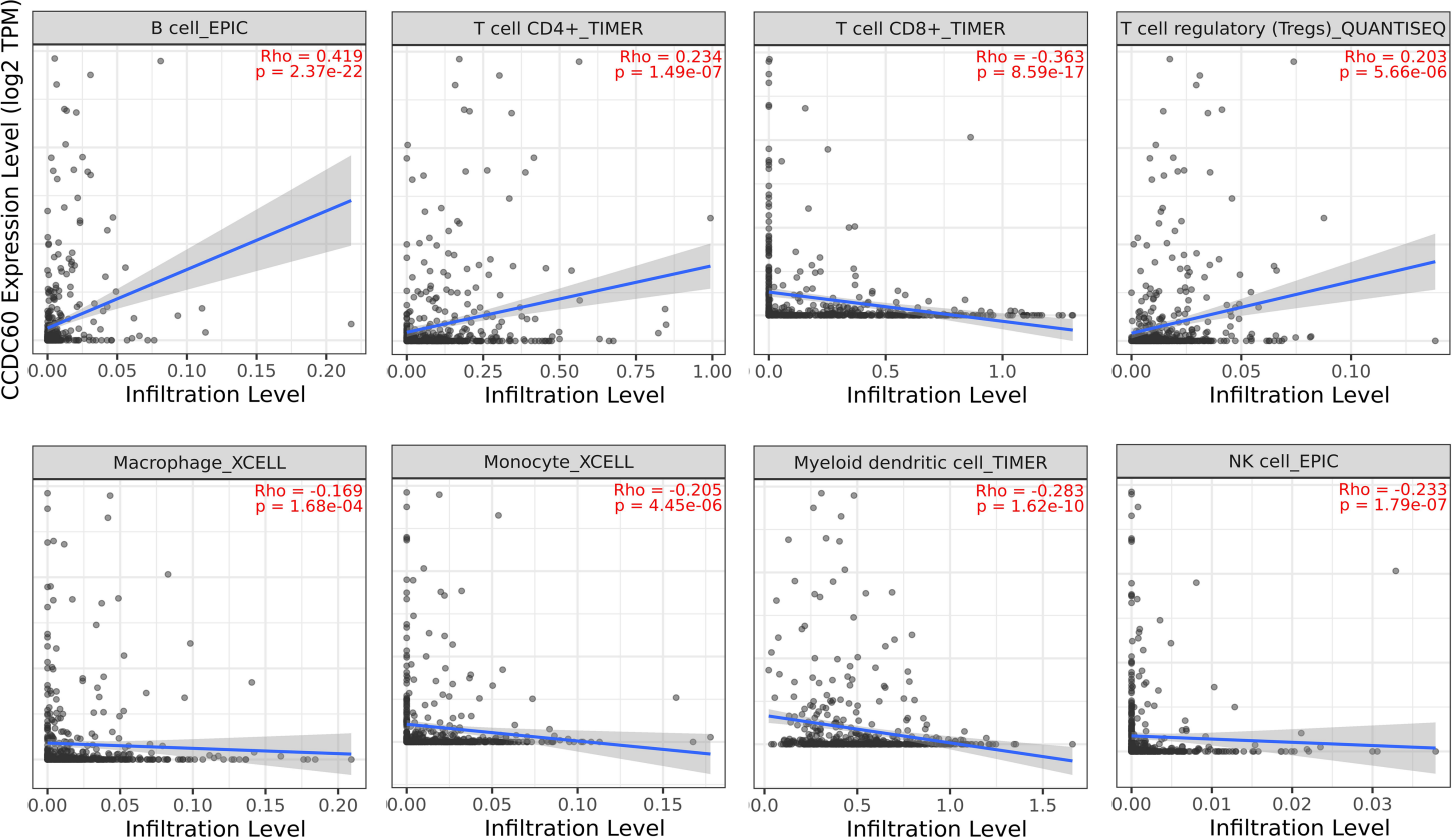
Relationship between CCDC60 expression and tumor-infiltrating lymphocytes in HNSC *via* the TIMER2.0 database.

Tumor-infiltrating lymphocytes (TILs), as an integral part of the tumor microenvironment, was correlated with the clinical outcomes and therapy responses in cancers. The above findings supported a prognostic role of CCDC60 in HNSC, but its function within the TME remained unknown. As definitely presented in **Figure 6**, CCDC60 expression was significantly positive linked with the infiltration levels of B cells (Rho = 0.419, P = 2.37e-22), CD4+ T cells (Rho = 0.234, P = 1.49e-07) and Tregs (Rho = 0.203, P = 5.66e-06). Furthermore, CCDC60 expression was negatively related to the infiltration levels of CD8+ T cells (Rho = -0.363, P = 8.59e-17), Macrophages (Rho = -0.169, P = 1.68e-04), Monocytes (Rho = -0.205, P = 4.45e-06), DCs (Rho = -0.283, P = 1.62e-10) and NK cells (Rho = -0.233, P = 1.79e-07). These analyses suggested that CCDC60 had a close connection with the immune infiltrating cells in HNSC, and further studies of their interactions and potential immune-related mechanisms are needed.

### 3.7 Correlation between CCDC60 expression and immune marker sets in HNSC

**Figure 7:**
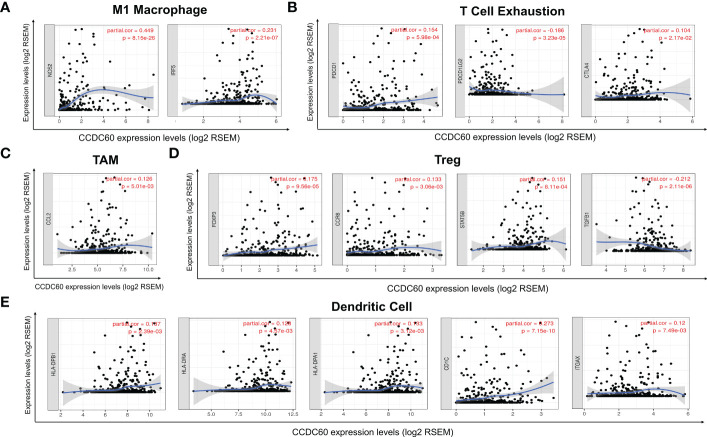
Correlation analysis of immune markers with CCDC60 expression in HNSC. Scatterplots of relationships between CCDC60 and gene markers of M1 Macrophage **(A)**, T Cell Exhaustion **(B)**, TAM **(C)**, Treg **(D)** and Dendritic Cell **(E)** in HNSC (n = 520).

Subsequently, our study explored the association based on the immune marker sets of TILs. Our analysis revealed that CCDC60 was closely correlated with the majority of gene markers of immune cells in HNSC (**Table 2**). We noticed that the expression levels of marker set of M1 macrophages (NOS2 and IRF5), TAMs (CCL2) had strong associations with CCDC60 expression in HNSC (**Figures 7A**, **C**). CCDC60 expression was significantly linked to T cell exhaustion markers (PDCD1, PDCDLG2 and CTLA4) in HNSC (**Figure 7B**), suggesting that CCDC60 may have an impact on the immune escape in HNSC. For Treg cells, CCDC60 showed significant correlation with FOXP3, CCR8, STAT5B and TGFB1 in HNSC (**Figure 7D**). High CCDC60 expression was significantly connected with the infiltration level of DCs in HNSC, DC markers such as HLA-DPB1, HLA-DRA, HLA-DPA1, CD1C and ITGAX were also significantly correlated with CCDC60 expression (**Figure 7E**). The above findings strongly suggested that CCDC60 played a key function in the regulation of immune infiltration in HNSC.

**Table 2:** Correlation analysis between CCDC60 and related genes and markers of immune cells in Tumor Immune Estimation Resource (TIMER2.0).

Description	Gene markers	HNSC			
		None		Purity	
		Cor	P	Cor	P
T cell (general)	CD3D	0.173	***	0.163	***
	CD3E	0.187	***	0.188	***
	CD2	0.185	***	0.183	***
CD4 + T cell	CD4	0.131	**	0.131	**
CD8 + T cell	CD8A	0.14	**	0.133	**
	CD8B	0.183	***	0.173	***
Th1	T -bet (TBX21)	0.138	**	0.123	**
	STAT4	0.086	*	0.084	0.0633
	STAT1	-0.109	*	-0.107	*
	IFN-γ (IFNG)	0.046	0.295	0.03	0.509
	TNF-α (TNF)	0.047	0.284	0.052	0.248
Th2	GATA3	0.111	*	0.115	*
	STAT6	0.171	***	0.182	***
	STAT5A	0.235	***	0.217	***
	IL13	0.065	0.137	0.078	0.0847
Tfh	BCL6	0.308	***	0.312	***
	IL21	0.172	***	0.165	***
Th17	STAT3	0.27	***	0.274	***
	IL17A	0.208	***	0.214	***
Treg	FOXP3	0.172	***	0.175	***
	CCR8	0.132	**	0.133	**
	STAT5B	0.162	***	0.151	***
	TGFβ (TGFB1)	-0.236	***	-0.212	***
T cell exhaustion	PD-1 (PDCD1)	0.156	***	0.154	***
	PDL1(PDCD1LG2)	-0.185	***	-0.186	***
	CTLA4	0.104	*	0.104	*
	LAG3	0.023	0.604	0.018	0.685
	TIM-3 (HAVCR2)	0.046	0.294	0.044	0.327
	GZMB	0.079	0.0696	0.075	0.0965
B cell	CD19	0.355	***	0.369	***
	CD79A	0.38	***	0.395	***
Monocyte	CD86	0.04	0.359	0.037	0.408
	CD115 (CSF1R)	0.075	0.0862	0.079	0.0789
Neutrophils	CD66b (CEACAM8)	0.216	***	0.206	***
	CD11b (ITGAM)	0.139	**	0.129	**
	CCR7	0.233	***	0.237	***
TAM	CCL2	0.13	**	0.126	**
	CD68	-0.038	0.384	-0.035	0.442
	IL10	0.01	0.828	0.017	0.713
M1 Macrophage	INOS (NOS2)	0.454	***	0.449	***
	IRF5	0.236	***	0.231	***
	COX2(PTGS2)	0.055	0.208	0.056	0.213
M2 Macrophage	CD163	-0.049	0.268	-0.035	0.436
	VSIG4	-0.002	0.972	0.012	0.785
	MS4A4A	-0.012	0.778	-0.009	0.85
Natural killer cell	KIR2DL1	0.047	0.28	0.05	0.266
	KIR2DL3	0.063	0.151	0.053	0.243
	KIR2DL4	0.096	*	0.101	*
	KIR3DL1	0.092	*	0.082	0.0691
	KIR3DL2	0.18	***	0.192	***
	KIR3DL3	0.09	*	0.09	*
	KIR2DS4	0.024	0.577	0.017	0.708
Dendritic cell	HLA-DPB1	0.138	**	0.137	**
	HLA-DQB1	0.08	0.0665	0.077	0.0864
	HLA-DRA	0.131	**	0.128	**
	HLA-DPA1	0.137	**	0.133	**
	BDCA-1(CD1C)	0.275	***	0.273	***
	BDCA-4(NRP1)	-0.04	0.362	-0.033	0.46
	CD11c (ITGAX)	0.111	*	0.12	**

HNSC, Head and Neck squamous cell carcinoma; TAM, tumor-associated macrophage; Th, T helper cell; Tfh, Follicular helper T cell; Treg, regulatory T cell; Cor, R value of Spearman’s correlation; None, correlation without adjustment; Purity; correlation adjusted by purity. *p < 0.01; **p < 0.001; ***p < 0.0001.

### 3.8 Correlation between CCDC60 expression and immunomodulators in HNSC

**Figure 8:**
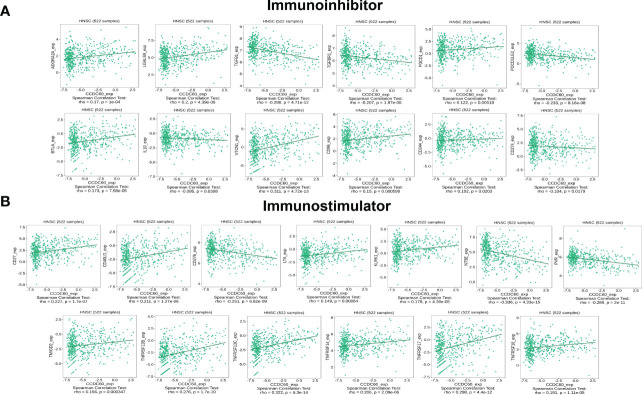
CCDC60 expression was correlated with immunomodulators in HNSC. **(A)** Relationship of CCDC60 expression with immunoinhibitors in HNSC samples using the TISIDB database. **(B)** Relationship of CCDC60 expression with immunostimulators in HNSC samples using the TISIDB database.

Our findings showed that CCDC60 expression was closely connected with immunoinhibitors (P < 0.05, **Figure 8A**), CCDC60 was positively related to ADORA2A (Rho = 0.170. P = 1.00e-04), LGALS9 (Rho = 0.200, P = 4.39e-06), PDCD1 (Rho = 0.122, P = 5.18e-03), BTLA (Rho = 0.173, P = 7.58e-05), VTCN1 (Rho = 0.311, P = 4.72e-13), CD96 (Rho = 0.150, P = 5.98e-04) and CD244 (Rho = 0.102, P =2.03e-02), while negatively correlated with TGFB1 (Rho = -0.298, P = 4.71e-12), TGFBR1 (Rho = -0.207, P = 1.87e-06), PDCD1LG2 (Rho = -0.233, P = 8.16e-08), IL-10 (Rho = -0.095, P = 3.08e-02) and CD274 (Rho = -0.104, P = 1.79e-02). Additionally, the expression of CCDC60 was significantly connected with immunostimulators (P < 0.001, **Figure 8B**). CCDC60 was positively correlated with CD27 (Rho = 0.227, P = 1.70e-07), CD40LG (Rho = 0.211, P = 1.27e-06), LTA (Rho = 0.149, P = 6.40e-04), KLRK1 (Rho = 0.178, P = 4.39e-05), TMIGD2 (Rho = 0.156, P = 3.47e-04), TNFRSF13B (Rho = 0.276, P = 1.70e-10), TNFRSF13C (Rho = 0.322, P = 6.30e-14), TNFRSF14 (Rho = 0.206, P = 2.09e-06), TNFRSF17 (Rho = 0.298, P = 4.40e-12) and TNFRSF18 (Rho = 0.191, P = 1.11e-05), while CD276 (Rho = -0.251, P = 6.62e-09), PVR (Rho = -0.298, P = 2.00e-11) and NT5E (Rho = -0.336, P = 4.33e-15) were negatively correlated. The above results reminded us that CCDC60 had a regulatory effect on the immune interactions and mediated tumor immune escape in HNSC, which could improve the response to immunotherapy for HNSC patients.

### 3.9 Correlation between CCDC60 expression and chemokines in HNSC

**Figure 9:**
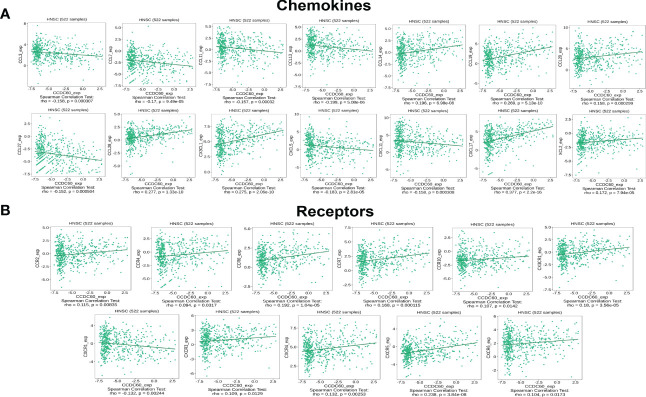
CCDC60 expression was correlated with chemokines in HNSC. **(A)** Relationship of CCDC60 expression with chemokines in HNSC samples using the TISIDB database. **(B)** Relationship of CCDC60 expression with chemokine receptors in HNSC samples using the TISIDB database.

Subsequently, we investigated the relationship between CCDC60 and chemokines in HNSC. We demonstrated that CCDC60 expression was positively linked with CCL14 (Rho = 0.196, P = 6.98e-06), CCL19 (Rho = 0.269, P = 5.13e-10), CCL20 (Rho = 0.158, P = 2.99e-04), CCL28 (Rho = 0.277, P = 1.33e-10), CX3CL1 (Rho = 0.275, P = 2.06e-10), CXCL17 (Rho = 0.377, P < 2.20e-16) and XCL2 (Rho = 0.172, P = 7.94e-05), and negatively with CCL3 (Rho = -0.158, P = 3.07e-04), CCL7 (Rho = -0.170, P = 9.49e-05), CCL11 (Rho = -0.157, P = 0.32e-03), CCL13 (Rho = -0.199, P = 5.08e-06), CCL27 (Rho = -0.152, P = 5.04e-04), CXCL5 (Rho = -0.183, P = 2.81e-05) and CXCL11 (Rho = -0.158, P = 3.08e-04) (P < 0.001, **Figure 9A**). We also found that the expression of CCDC60 was strongly related to chemokine receptors (P < 0.05, **Figure 9B**). Except CXCR1 (Rho = -0.132, P = 2.44e-03), the expression of other chemokine receptors was positively correlated with CCDC60, including CCR2 (Rho = 0.115, P = 8.35e-03), CCR4 (Rho = 0.094, P = 3.17e-02), CCR6 (Rho = 0.192, P = 1.04e-05), CCR7 (Rho = 0.168, P = 1.15e-04), CCR10 (Rho = 0.107, P = 1.42e-02), CX3CR1 (Rho = 0.180, P = 3.56e-05), CXCR3 (Rho = 0.109, P = 1.29e-02), CXCR4 (Rho = 0.132, P = 2.53e-03), CXCR5 (Rho = 0.238, P = 3.84e-08), CXCR6 (Rho = 0.104, P = 1.73e-02). This study revealed the close relationships between CCDC60 expression and chemokines as well as chemokine receptors and further proved that CCDC60 may be a promising immunomodulatory factor in HNSC.

## 4 Discussion

Head and neck cancers is the seventh most common malignant tumors around the world, with a high risk of recurrence and metastasis and a very poor prognosis (25). The early diagnosis of HNSC patients is very difficult due to the lack of obvious clinical symptoms. At present, the search for new biomarkers and elucidating the underlying molecular mechanisms are important directions for the early diagnosis of HNSC. CCDC60 is a member of CCDC proteins family, which presents huge potential to inhibit tumor growth and mediate biological processes in many cancers, such as bladder cancer and gastric cancer (26, 27). However, the effect of CCDC60 on the prognosis and immunomodulation of HNSC remains unclear. Our study comprehensively revealed the key function of CCDC60, which could be used as a diagnostic and prognostic indicator related to the regulation of immune infiltration and improvement of the response to immunotherapy in HNSC patients.

First, we applied the TIMER2.0 database to conduct pan-cancer analysis on the expression of CCDC60, and further confirmed the downregulated CCDC60 expression in HNSC tissues using the TCGA database (n= 546), compared with the healthy samples. Our analyses revealed that CCDC60 expression was widely related to individual cancer stage, lymph node metastasis status, tumor grade, TP53 status, patient’s gender and age of HNSC patients. These results were consistent with those from qRT-PCR in 23 pairs of clinical HNSC samples with or without lymph node metastasis. The analysis of ROC curve indicated that CCDC60 gene had a good predictive performance for HNSC diagnosis (AUC = 0.807), indicating that CCDC60 may be a valuable diagnostic biomarker for HNSC patients. The results of survival analysis suggested that upregulated CCDC60 expression was significantly linked with better OS and longer DSS in HNSC patients. Moreover, increased CCDC60 expression was significantly connected with a better prognosis of HNSC patients in grade 3, stages 2 to 4 with the highest HR for longer OS. The above findings have showed that CCDC60 may be an indicator linked with diagnosis and prognosis, and the prognostic significance of CCDC60 depended on its distinct clinical characteristics in HNSC.

Researches have suggested that genetic alterations were closely associated with tumorigenesis and had an impact on the prognosis of cancers (28). We further evaluated the alteration frequency and alteration-related prognosis of CCDC60 in HNSC samples by cBioPortal database. The genetic alteration of CCDC60 (~1.8%, frequency) mainly occurred in amplification and mutation in HNSC samples, and the diploid was the most common copy number variations; however, we could not find the significant relationship between CCDC60 alteration and the prognosis of HNSC. Subsequently, we identified the CCDC60 related co-expressed genes and GSEA analysis revealed that these genes were mainly enriched in the NOD-like receptor signaling pathway, associated with chemical carcinogenesis. Previously studies have demonstrated that NOD-like receptor (NLR) served as an anti-oncogene, and was involved in the process of head and neck cancers (29). NLRP3 was correlated with the tumor growth and metastasis of oral squamous cell carcinoma (OSCC), and knockdown of NLRP3 significantly inhibited the proliferation, migration and invasion of OSCC cells (30). We further detected the impact of CCDC60 on the biological behavior of HNSC cells, *in vitro*. Functional assays revealed that CCDC60 not only significantly inhibited the growth, migration and invasiveness but regulated cell cycle progression and promoted cell adhesion of Fadu and Cal27 cells. These experimental data confirmed the results of bioinformatics analysis, suggesting that CCDC60 played a tumor suppressor role through the NOD-like receptor signaling pathway, leading to a longer prognosis of HNSC patients.

Another focus of our study was on the function of CCDC60 in tumor immunity and its potential mechanism of immune regulation in HNSC. Tumor-infiltrating lymphocytes (TILs) had a significant influence on the tumorigenesis, and were closely related to the prognosis and the response to immunotherapy of different cancers (31). We examined the relation of CCDC60 expression and immune infiltration in HNSC, and analysis suggested that CCDC60 was strongly connected with the abundance of TILs. The expression of CCDC60 was significantly positive linked with the infiltrating levels of B cells, CD4+ T cells and Tregs, while negatively corelated with Macrophages, Monocytes, DCs and NK cells in HNSC. Additionally, the connection of CCDC60 expression with immune marker sets of TILs suggested the vital role of CCDC60 in regulating immunity of HNSC. Studies have shown that the polarization state of TAMs and its proportion in TME could significantly affect tumor growth, invasion and metastasis (32, 33). We found CCDC60 expression was correlated with TAM markers (CCL2) and M1 macrophage markers (NOS2 and IRF5), which reminded us that CCDC60 may regulate HNSC progression by influencing macrophage polarization. Won et al. demonstrated that FOXP3 gene played a tumor suppressor in lung squamous cell carcinoma (34). and it had been shown that DCs could modulate tumor metastasis by improving Tregs responses and reducing CD8+ T cell cytotoxicity (35). The expression of CCDC60 had a close correlation with the Treg markers (FOXP3, CCR8, STAT5B and TGFB1) and DCs markers (HLA-DPB1, HLA-DRA, HLA-DPA1, CD1C and ITGAX), suggesting CCDC60 may be involved in the progression and metastasis of HNSC by mediating DCs infiltration and Tregs responses. Moreover, high CCDC60 expression was significantly linked to T cell exhaustion markers (PDCD1, PDCDLG2 and CTLA4) in HNSC, suggesting that CCDC60 may have an impact on the immune escape in HNSC (36). Consisting of immunoinhibitors and immunostimulators, immunomodulators played a key function in modulating the function of immune system, and were considered as promising approaches for cancer immunotherapy (37). Studies have shown that chemokines could significantly affect tumorigenesis, tumor immunity, and control the infiltration degree of immune cells (38). Nowadays, the PD-1/PD-L1 checkpoint blockade immunotherapy showed satisfactory efficacy as well as low toxicity in advanced HNSC patients; however, due to the important role of PD-1 in tumor antigen tolerance, the curative effects of PD-1 therapy in some patients was poor (39, 40). Therefore, it was necessary to enhance the tumor cells response to immune checkpoint inhibitors and cytokines. Based on the TISIDB and TIMER2.0 databases, we realized that up-regulated CCDC60 expression was linked with immunomodulators such as PD-1/PD-L1 and CTLA4, and significantly linked with cell responses to chemokines, which would be a promising strategy to raise the efficacy of immunotherapy by targeting CCDC60. Together these findings suggest that CCDC60 might regulate the immune infiltration and improve the response to immunotherapy in HNSC patients, which has an important effect on the tumor microenvironment of HNSC. And CCDC60 could be identified as a novel predictor of HNSC immunotherapy response, leading to new therapeutic approaches that may modify their courses and improve the efficacy of HNSC immunotherapy.

Our research provides novel insights into promising biomarker for predicting response to HNSC immunotherapy and elucidate the potential mechanism of immune regulation behind it. This study found that CCDC60 can be used to select patients who are sensitive to targeted therapy or immunotherapy, and its diagnostic, therapeutic, and prognostic effects can enhance the impact on HNSC patients. However, there are some limitations to our study. There are relatively few clinical HNSC samples, so more patients need to be recruited for analysis. In addition, our study lacks the information about patients’ complications, treatment option, and so on. Finally, the potential immune regulation mechanism related to CCDC60 gene need to be further studied.

## 5 Conclusion

As a tumor suppressor gene, CCDC60 was related to a longer prognosis and involved in regulating the immune infiltration and enhancing the response to immunotherapy of HNSC patients. With high sensitivity, specificity, and accuracy, CCDC60 is able to identify HNSC patients who may really benefit from treatment with immunotherapy, and would allow to refine the therapeutic options and to better adjust the treatment strategies.

## Data availability statement

The original contributions presented in the study are included in the article/**Supplementary Material**. Further inquiries can be directed to the corresponding author.

## Ethics statement

This study was approved by the Institutional Ethics Committee at Qilu Hospital of Shandong University. The patients/participants provided their written informed consent to participate in this study.

## Author contributions

HL designed this study. ZL and SC wrote the paper and performed the bioinformatics analysis of the study. SC, WJ and YQ carried out the experiment based on HNSC tissues and HNSC cell lines. MZ and TF collected the samples and did statistical analysis. All authors contributed to the article and approved the submitted version.

## Supplementary material

Supplementary Material 1 HNSCC patients’ detailed clinical information.

Supplementary Material 2 Datasets of HNSC samples from TCGA database.

Supplementary Material 3 Data of CCDC60 related co-expressed genes in HNSC.

Supplementary Figure 1 Successful transfection of pENTER-CCDC60 overexpressed plasmid **(A)** anti-CCDC60 and **(B)** anti-FLAG.
