# Supplementary figures and images for: Comprehensive analysis reveals CCDC60 as a potential biomarker correlated with prognosis and immune infiltration of head and neck squamous cell carcinoma
Source: Front Oncol. 2023 Mar 30;13:1113781. doi: 10.3389/fonc.2023.1113781 (PMC10098326; doi:10.3389/fonc.2023.1113781)

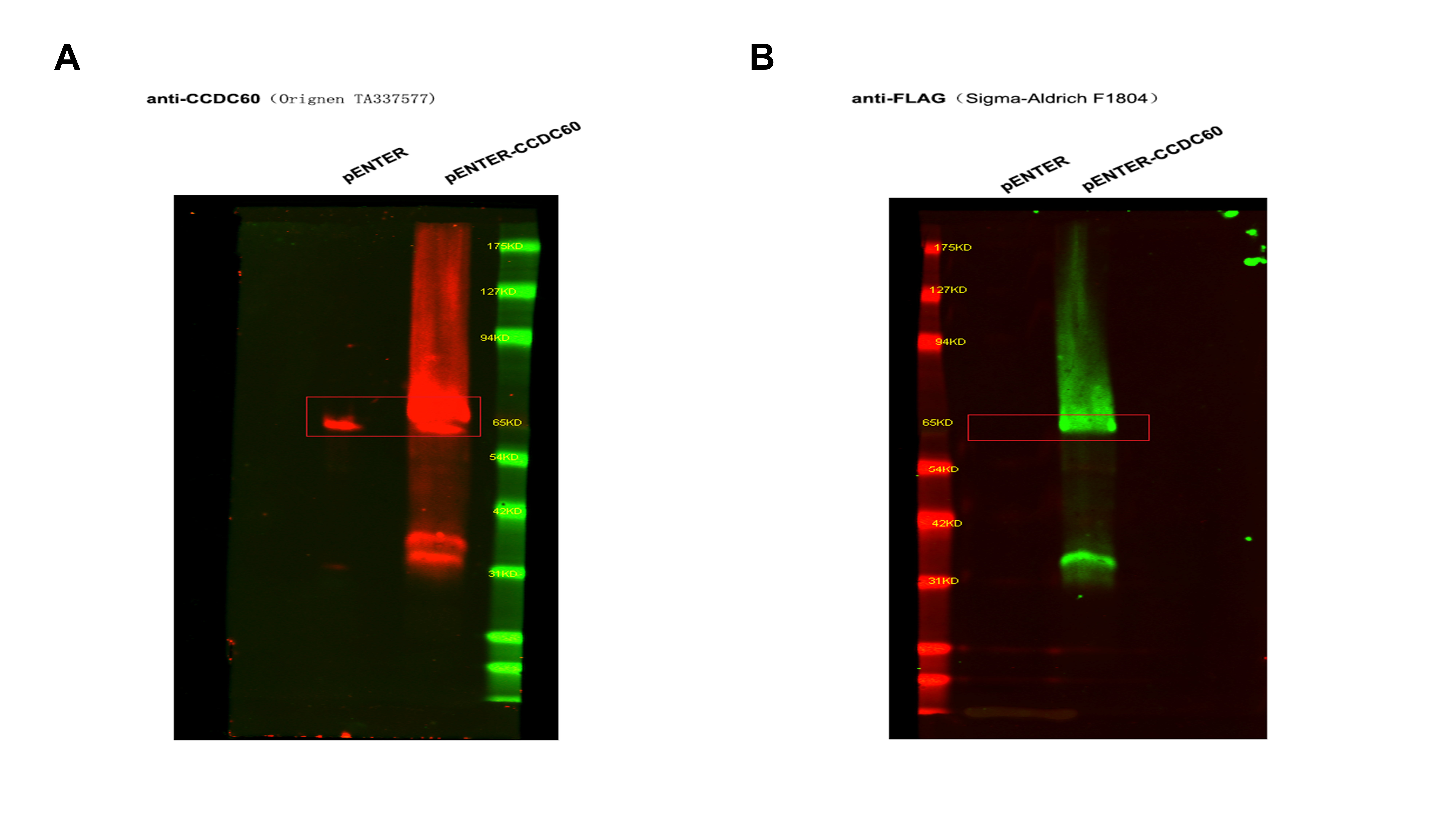

Supplement: Supplementary Figure 1 — Successful transfection of pENTER-CCDC60 overexpressed plasmid (A) anti-CCDC60 and (B) anti-FLAG. [file Image_1.tif]
